# Association of the Gelatinase B/Metalloproteinase 9 (MMP-9) Gene Haplotype in Systemic Lupus Erythematosus (SLE) in the Pediatric Egyptian Population

**DOI:** 10.3390/children9091271

**Published:** 2022-08-24

**Authors:** Zeinab R. Attia, Mohamed M. Zedan, Thuraya M. Mutawi, Entsar A. Saad, Rania A. Abd El Azeem, Mohamed A. El Basuni

**Affiliations:** 1 Mansoura University Children’s Hospital, Mansoura University, Mansoura 35516, Egypt; 2 Department of Pediatrics, Faculty of Medicine, Mansoura University, Mansoura 35516, Egypt; 3 Chemistry Department, Faculty of Science, Damietta University, Damietta 34511, Egypt

**Keywords:** SNPs, haplotypes, inflammation, matrix metalloproteinase-9, SLE risk, genotyping, Egyptian children and adolescents

## Abstract

Permanent systemic inflammation is a defining feature of systemic lupus erythematosus (SLE), which affects multiple organs. Gelatinase B/matrix metalloproteinase-9 (MMP-9) is an essential protease investigated in inflammation that has been linked to SLE. The study’s objective was to investigate the relationship between the rs3918249 T/C and rs17576 A/G SNPs in the MMP-9 gene with SLE. The study was conducted with 100 SLE cases and 100 age/sex-matched healthy individuals. TaqMan^TM^ SNP was used for genotyping by real time PCR on the Artus Rotor-Gene Qiagen equipment. Haplotypes (TG: OR = 0.226, 95% CI = 0.119–0.429) and (CA: OR = 0.36, 95% CI = 0.2206–0.631), both with a *p*-value < 0.001 were substantially linked to a lower incidence of SLE. Conversely, the risk of SLE was not associated with the individual SNPs studied. The haplotype analysis was more significant than the SNP analysis and may correlate with the decreased risk of SLE in children and adolescents in Egypt.

## 1. Introduction

It is generally known that inflammatory response contributes to the onset, development, and expression of several organ disorders. For example, it contributes to nephropathies [1], severe renal failure [2,3], liver cell dysfunction [4], and systemic lupus erythematosus (SLE) disease [5,6]. The latter is marked by permanent systemic inflammation and implicates several organs [5,6,7]; it reveals numerous clinical manifestations, together with immune complex sedimentations in kidneys and other organs. It is characterized by the formation of a wide range of autoantibodies versus various antigens (nuclear, cytoplasmic, and cell surface), and various deteriorations of the functioning of B-lymphocyte and T-cell [8]. In addition to the impacts of inflammatory mediators, activation of MMP-2 and MMP-9 has been demonstrated to be a major factor in the development of SLE [5].

The family of Zn2+-dependent endoproteases known as matrix metalloproteinases (MMPs) has a variety of roles in tissue remodeling [9]. They are involved in degrading extracellular matrix proteins such as fibronectin, a key part of extracellular matrix proteins linked to several different forms of human cancer [10], and they take part in extracellular tissue remodeling and degeneration in several liver disorders [11], many cancers, and arthritis causes joint destruction [12]. Numerous cells, including fibroblasts, vascular smooth muscle, and WBCs, release them. In addition to controlling many cellular and signaling pathways, they may modify the release or activation of chemokines, cytokines, and other bioactive molecules on the cell surface. They cause cell migration, proliferation, and differentiation and can affect apoptosis, inflammation, innate and acquired immunity, angiogenesis, and tissue repair [9,13]. Numerous growth factors, cytokines, and chemokines influence the transcription of several MMPs [14].

The matrix metalloproteinase-9 (MMP-9), also known as Gelatinase B/92-kDa type IV collagenase, is the biggest member of the gelatinases group in the MMPs family, and it works as a pro-inflammatory factor [15]. A gene on the long arm (q11–q13) of the 20th chromosome encodes gelatinase B/MMP-9. It is a significant enzyme examined in SLE [16] and was found to be closely related to SLE pathogenesis [8,17]. It is proposed to subscribe to vascular wall damage [18] and increased risk of ischemia–reperfusion actions in SLE neuropathy [19]. Genetic variations of Gelatinase B may modify its expression resulting in inflammatory diseases. The most common widely studied Gelatinase B/MMP-9 SNPs in different populations include rs3918242, rs3918254, rs2250889, rs3918249, and rs17576 [20]. The SNP rs3918249 is located in intron 1, and functional polymorphism rs17576 is located in exon 6 [21,22].

Till now, there has been no evidence available on the association degree of Gelatinase B/MMP-9 rs3918249 T/C and rs17576 A/G SNPs with SLE disease severity in Egyptian kids and youths. This study was conducted to learn more about the genotypes of these two SNPs, their haplotypes, and how they relate to how severe SLE is in Egyptian people.

## 2. Materials and Methods

### 2.1. Participants in the Study

There were 100 unconnected children with SLE included in this cross-sectional study; with a median age of 13.8, plus or minus 2.9 years, and a disease that persisted for 3.6 ± 2.9 years. One hundred healthy, unrelated, matched-age, matched-gender Egyptian youngsters were used as controls; they had no inflammatory or autoimmune diseases. In Table 1, the SLE patients’ clinical features are listed. SLEDAI calculated disease progress (Systemic Lupus Erythematosus Disease Activity Index) [23]. They were chosen from the Mansoura University Children’s Hospital in Egypt’s pediatric nephrology clinic. The Mansoura University Faculty of Medicine’s Institutional Review Board (IRB), made recommendations for how to obtain written informed parental agreement, as well as child assent from participants. The certificate of ethical approbation is (R.20.09.1020). The Declaration of Helsinki’s guidelines served as the study’s direction.

### 2.2. Collection and Processing of Samples

Blood samples from both patients and controls were taken. Three separate blood samples were drawn: 1000 µL into an ethylenediaminetetraacetic acid (EDTA) vial for extraction of DNA, 1000 µL into an EDTA tube for a full blood count, and 3000 µL into a transparent plastic collection tube. After allowing the blood to coagulate, it was centrifuged for 10 min at room temperature at 3000 rpm. The serum was then kept at 2–8 °C for up to 5 days before needed and stored at −20 °C or −80 °C for up to 6 months.

Following the manufacturer’s instructions, a manual sandwich ELISA kit (Cat. No. E0936Hu, Bioassay Technology Laboratory, Shanghai, China) was used to determine the amount of serum MMP-9 in patients and healthy children. While serum C3 and C4 were evaluated by a turbidimetric assay (DIALAB GmbH, Neudorf, Austria), immunological tests (Anti-Nuclear Antibodies (ANA) and Anti-dsDNA were assessed by ELISA technique utilizing Euroimmun, Germany, and INOVA Diagnostic, San Diego, CA, USA, respectively. Following the directions on their kit, the serum concentrations of phosphorus (SPINREACT, Girona, Spain), calcium, and creatinine (DIALAB, Neudorf, Austria) were measured.

### 2.3. Genetic Testing and DNA Extraction

The Qiagen QIAamp DNA kit (Germany) was used to extract genomic DNA in blood using the usual release methodology. To determine allelic variants of SNPs rs3918249 and rs17576 in the Gelatinase B/MMP-9 gene, a test for allelic discrimination was employed with precise TaqMan probes (ID: C_1414756_10 for rs3918249 T/C, C_11655953_10 for rs17576 A/G) utilizing the Artus Rotor-Gene Qiagen rapid real-time (RT) PCR System. (Applied Biosystems, Foster city, CA, USA, software 2.1.0). The PCR reaction will be carried out in a final volume of 20 μL per reaction, with 20 ng of extracted DNA and 10 μL TaqManTM Genotyping Master Mix (Thermo Fisher Scientific as the manufacturer’s protocol, Third Avenue. Waltham, MA USA). The PCR process started at 95 degrees for 10 min, then went through 45 cycles of 95 degrees for 15 s, and 60 degrees for 60 s.

### 2.4. Statistical Methods

The mean and standard deviation were used to depict numerical data (SD). The statistical significance of the difference between the means of the two study groups was evaluated using the Student’s *t*-test. To investigate the connection between two qualitative variables, the chi-square test was performed. The area under the curve (AUC) was measured from the receiver operating characteristic (ROC) curve. When the predicted count was less than five in more than 20% of cells, Fisher’s exact test was employed to investigate the link between two qualitative variables. The chi-square test was used to evaluate how deviations from Hardy–Weinberg equilibrium expectations differed. Using generalized linear models, logistic regression analysis was utilized to predict risk factors. The linkage disequilibrium (LD) and haplotypes were estimated using the HaploView tool (version 4.2) [24], which uses the expectation-maximization algorithm. The *p*-value is significant if <0.05 at a confidence interval (CI) of 95%. A Statistical Package for Social Science (SPSS) program was used for these analyses (IBM Corp. Released 2017. IBM SPSS Statistics for Windows, Version 25.0. IBM Corp, Armonk, NY, USA). According to Zhao et al., additive, dominant, and allelic models were outlined in our work. For example, if the gene of interest has two haploid alleles, A and B, and A is the “risk” or “increasing” allele, meaning it causes the effect, the three genotype groups would be AA, AB, and BB. [25]. This dichotomization of the SNP genotypes can be performed as follows:

- Dominant: “AA + AB” versus “BB”
- Additive: “AA” versus “AB” versus “BB”

In addition to the allelic model

- Allelic: A versus B

## 3. Results

### 3.1. The Study Population’s Clinical Manifestations

Table 1 illustrates that SLE patients were characterized by a heterogeneous collection of clinical manifestations. The rate of prevalence of these manifestations was highly variable. Manifestations with a low rate of prevalence appeared in 3–13% of SLE cases, including serositis, oropharyngeal ulcers, and neurologic conditions. Manifestations with a medium rate of prevalence appeared in 27–53% of cases, including arthritis, non-scarring alopecia, and skin disorders. Manifestations with a high rate of prevalence appeared in 72–100% of cases, including kidney, blood, and immunologic conditions.

SLE patients showed substantial drops in white blood cells (WBCs) and platelet count, hemoglobin level, C3, and C4 complement levels but they did not display significant changes in P, Ca, and creatinine levels (Table 2). All control subjects (100%) showed negative ANA and negative ds-DNA titer while 92% and 68% of SLE subjects showed positive ANA and positive ds-DNA titer, respectively.

### 3.2. Gelatinase B/MMP-9 Determination in Serum

**Figure 1:**
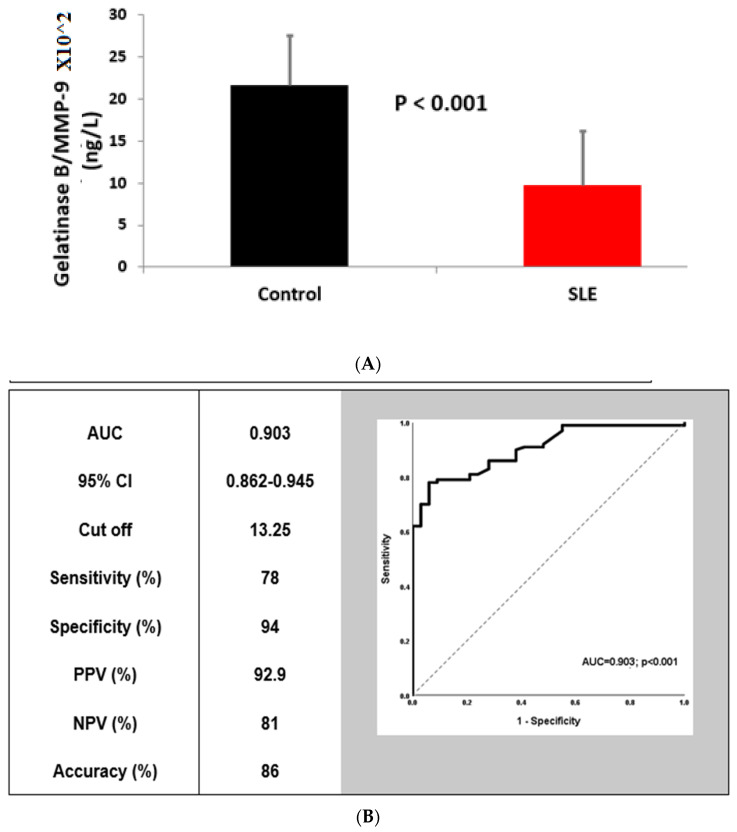
Gelatinase B/MMP-9 (**A**) level (ng/L) in SLE cases and control groups, and (**B**) receiver operating characteristic (ROC) curve of Gelatinase B/MMP-9 level for discrimination between SLE cases and healthy control subjects. Data are expressed as mean ± SD, *p* < 0.05 versus the control group is considered significant. Abbreviations: PPV stands for positive predictive value; NPV stands for negative predictive value; CI is for confidence interval, AUC: areas under curve.

Serum Gelatinase B/MMP-9 levels in the SLE group were substantially decreases compared to the controls (*p* less than 0.001 as shown in Table 2, Figure 1A,B). In SLE patients, the mean circulating levels of Gelatinase B/MMP-9 were inversely linked with the titer of ANA (*p* = 0.005, r = −0.278) and ds-DNA (*p* < 0.001, r = −0.376). However, there was no connection between serum levels of gelatinase B/MMP-9 and SLEDAI score (not illustrated in tables), proving that the disease activity was not related to the decreased serum levels of MMP-9. For discrimination between SLE cases and control groups, Gelatinase B/MMP-9 demonstrated an excellent AUC value extremely close to one (0.903) (Figure 1).

### 3.3. Association of Gelatinase B/MMP-9 rs3918249 T/C and rs17576 A/G Genotypes, Alleles, or Haplotypes with SLE Risk

Table 3 lists the genotype and allele frequencies of the tested SNPs among participants in the case and control groups. In addition, the genotype frequencies of Gelatinase B/MMP-9 rs3918249 T/C and rs17576 A/G in controls or SLE patients followed Hardy–Weinberg equilibrium. No significant association between any of Gelatinase B/MMP-9 rs3918249 T/C and rs17576 A/G genotypes or alleles with SLE risk.

We further assessed the relationship between the risk of getting SLE and the Gelatinase B/MMP-9 rs3918249 and rs17576 haplotype. Haplotypes “CA” and “TG” were highly significantly correlated with a lower risk of SLE (OR = 0.36, 95% CI; 0.206–0.631, *p* < 0.001) and (OR = 0.226, 95% CI (confidence interval; 0.119–0.429, *p* < 0.001), respectively (Table 4).

No significant associations of either polymorphism rs3918249 T/C or rs17576 A/G with SLE clinical features, SLEDAI, and MMP-9 levels were detected as illustrated in Table 5. Furthermore, none of the examined SLE biochemical indicators showed a significant correlation between circulating gelatinase B/MMP-9 levels (not illustrated in tables).

## 4. Discussion

SLE has been linked with many diverse genes. The majority of these connections still do not exist in different groups [26]. There is no genetic research reported from Egypt, which has examined this aspect of the Gelatinase B/MMP-9 SNPs. We discovered evidence of a possible haplotype link between SLE and these two SNPs in the MMP-9 gene.

In the present study, there is a wide group of variable manifestations, connected to different organs and tissues, which were not equally shown among the studied SLE cases. The immunological and hematological problems were of the highest prevalence, and they were practically confirmed here via the positive titer of anti-nuclear and anti-ds-DNA antibodies as well as the noticed declines in C3 and C4 levels, hemoglobin content, and blood cell count among SLE cases. Similarly, Bashal announced blood conditions as the most common features in SLE [27]. These findings, inconsistent with other researchers such as Qu et al. and Zhang et al. confirm autoantibodies as the chief contributors to SLE [28,29].

Despite the pathogenesis of SLE still not being entirely understood, investigations have pointed out its connection with deregulated MMPs [30]. In specific, Gelatinase B/MMP-9 involvement in SLE as a dangerous or advantageous molecule is still debatable because of contradictions in the published studies. Researchers reported varying amounts of circulating gelatinase B/MMP-9 in SLE patients and controls. Some reported higher levels, while others reported lower levels. However, others reported no changes [16]. Here in our Egyptian SLE cohort, the levels of serum Gelatinase B/MMP-9 were discovered to be dramatically decreased. This finding is compatible with other studies carried out on Iranian, Poland, Saudi Arabian, Chinese, and Korean SLE cases [5,31,32,33,34]. Whereas it is incompatible with other researchers e.g., Ram et al. and Faber-Elmann et al. who reported elevated Gelatinase B/MMP-9 in SLE patients’ sera [8,18]. Nevertheless, according to Mao et al., there was no appreciable difference in blood MMP-9 levels between SLE patients and healthy controls [17]. Despite these fluctuations decreased MMP-9 fabrication has been suggested as a potential genetic risk factor associated with unusually elevated immunological reactivity [34].

Although the reasons for the decreased MMP-9 serum levels in SLE are unknown, non-genetic elements including sample collecting techniques [35] or the number of peripheral blood neutrophils, the main source of serum MMP-9, can influence total serum concentrations [36]. Our current findings showed that circulating MMP-9 levels and both ANA and anti-ds-DNA antibodies had substantial negative relationships, which are specific markers of SLE, but not with SLEDAI. We hypothesized that the advanced SLE disease activity was not caused by the decreased serum levels of MMP-9. Our data are hand in hand with Makowski and Ramsby who found that MMP-9 levels and anti-ds-DNA levels showed an inverse association [37], and with Faber-Elmann et al. who did not see any connection between the blood MMP-9 level and SLEDAI that was significant [18], but it is in contradiction with Ahmad et al. and Liu et al., who demonstrated a negative correlation between MMP-9 serum level and SLEDAI [32,33]. In the current work, the ROC curve pointed out that circulated MMP-9 had an excellent power to discriminate SLE patients from healthy people (AUC = 0.903, accuracy = 86%, and *p* < 0.001).

Gelatinase B/MMP-9 rs17576 SNP was reported to have an association with many different diseases such as cancer, Henoch–Schonlein purpura, internal carotid artery bulb, and Behçet’s disease [15]. While Gelatinase B/MMP-9 rs3918249 SNP was connected to asthma and glaucoma [38]. In our work, we demonstrated that neither the rs3918249 T/C nor rs17576 A/G SNPs in the Gelatinase B/MMP-9 gene were linked to SLE susceptibility in Egyptian populations. We also carried out a haplotype analysis because association studies have shown it to be a more reliable technique. Upon our current work findings, a most likely beneficial role of Gelatinase B/MMP-9 in SLE is postulated. We did not catch a significant association between rs3918249 as well as rs17576 genotypes and alleles and the risk of SLE development. Nevertheless, rs3918249–rs17576 haplotype frequency was calculated. “CG” haplotype showed the lowest frequency in controls while the “TG” haplotype showed the lowest frequency in cases. Considering “TA” as the reference haplotype, “CA” and “TG” were considered protective haplotypes against SLE development. Therefore, patients carrying these haplotypes (“CA” and “TG”) have a decreased risk of SLE susceptibility. Our findings are consistent with those of Ugarte-Berzal et al. who demonstrated that the MMP-9 has a beneficial role in SLE [16]. Moreover, the protective role of Gelatinase B/MMP-9 haplotype has been demonstrated by Gao et al. among the Chinese Han population [38], and by La Russa et al. in Italian multiple sclerosis patients [21]. These findings provide credence to the idea that the Gelatinase B/MMP-9 gene has a significant protective function in illnesses. This is the first study that we are aware of that looks at MMP-9 gene haplotypes with SLE illness in Egypt.

According to our research, there is no correlation between the clinical manifestations of SLE and the Gelatinase B/MMP-9 rs3918249 T/C and rs17576 A/G genotype frequencies. There is no connection between MMP-9 SNPs and clinical symptoms, according to a study conducted on Korean SLE patients to evaluate the association between MMP-9 promoter rs3918242 C/T and −90 (CA) (*n*) repeat polymorphisms on the clinical manifestations [34]. While it was discovered that the MMP-9 rs3918242 C/T genotype was linked to the clinical symptoms of SLE in the hematological (*p* = 0.02) and renal (*p* = 0.008) domains, [39].

Our findings showed that neither the rs3918249 T/C genotype nor the rs17576 A/G genotype significantly affected the levels of circulating MMP-9 indicating that serum levels and genetic polymorphisms of Gelatinase B/MMP-9 were independent of each other. Demacq et al. found that their genetic polymorphisms did not affect the serum MMP-9 concentrations [40]. On the contrary, Grzela et al. assumed that MMP-9 activity might be attributed to some variations in its molecular structure that are corresponding to some of its genetic SNPs [41]. These variations may be explained by racial heterogeneity and may also be caused by different populations’ exposure to diverse environmental factors.

The current state of our research has some limitations. Since the sample size of the patients under investigation was so tiny, large sample studies are necessary. Additionally, all of the patients in this study were from the Delta region of Egypt. To evaluate the impact of people’s residence on their results, we were unable to locate instances from other locations to include in our study. We only examined two genetic variations, as well. More study of the Gelatinase B/MMP-9 gene variations is required to comprehend the role of this gene in SLE.

## 5. Conclusions

Our study showed that the risk of SLE was not affected by the individual SNPs studied in the MMP-9 gene but was correlated with the two-locus haplotypes investigated in the same gene. Furthermore, the “CA” and “TG” (rs3918249 T/C- rs17576 A/G) haplotypes were associated with a reduced risk of SLE in children and adolescents from Egypt. It will take more research with a larger sample size to verify these results.

## Figures and Tables

**Table 1:** Clinical and demographic characteristics of the healthy control subjects and SLE (systemic lupus erythematosus) patients.

	SLE Patients	Healthy Control
Number	100	100
Sex ratio (female/male) (%)	94/6	93/7
Age (y) (mean ± SD)	(13.8 ± 2.9)	(13.5 ± 1.9)
Duration of the disease (y) (mean ± SD)	(3.6 ± 2.9)	n. a.
SLEDAI median (minimum–maximum)	8 (0–19)	n. a.
Active (SLEDAI > 10) (%)	49	n. a.
Inactive (SLEDAI < 10) (%)	51	
Clinical features		
Serositis (pleurisy/pericarditis) (%)	3	n. a.
Oropharyngeal ulcers (%)	10	n. a.
Neurologic disorders (%)	13	n. a.
Non-scarring alopecia (%)	31	n. a.
Arthritis (%)	27	n. a.
Photosensitivity (%)	32	n. a.
Malar rash (%)	39	n. a.
Laboratory findings		
complement components (C3/C4) (%)	97	n. a.
Anti-ds-DNA antibodies (%)	68	n. a.
Antinuclear antibody (%)	92	n. a.
Hemolytic Anemia (%)	64	n. a.
Thrombocytopenia (%)	12	n. a.
Leucopenia/lymphopenia (%)	10	n. a.
Pancytopenia (%)	5	n. a.
Persistent proteinuria (%)	72	n. a.
RBCs casts (%)	23	n. a.

Notes: values reported as median (range), percentile, and mean SD. Abbreviations: y: year, n. a: not applicable, SLE: systemic lupus erythematosus, SLEDAI: Systemic Lupus Erythematosus Disease Activity Index, Anti-ds-DNA: anti-double-stranded DNA, RBCs: red blood cells.

**Table 2:** Laboratory investigations of SLE (systemic lupus erythematosus) patients and healthy control subjects.

Studied Parameters	Control (*n* = 100)	SLE (*n* = 100)	*p* Values
White blood cells (×10^9^/L) (M± SD)	(8.7 ± 1.5)	(6.6 ± 1.6)	0.003
Red blood cells (×10^6^/L) (M ± SD)	(4.9 ± 0.4)	(4.5 ± 0.6)	0.032
Hemoglobin (g/dL) (M ± SD)	(12.9 ± 1.8)	(11.7 ± 2.8)	0.004
Platelets (×10^9^/L) (M ± SD)	(394.3 ± 66.2)	(282.1 ± 91.4)	<0.001
Creatinine (mg/dL) (M ± SD)	(0.7 ± 0.1)	(0.7 ± 0.2)	0.231
Phosphorus (mg/dL) (M ± SD)	(4.8 ± 0.5)	(4.9 ± 0.7)	0.272
Calcium (mg/dL) (M ± SD)	(9.2 ± 0.4)	(9.4 ± 0.7)	0.462
Complement 3 (mg/dL) (M ± SD)	(121.3 ± 18.1)	(107.9 ± 35.4)	0.001
Complement 4 (mg/dL) (M ± SD)	(22.4 ± 6.8)	14.1	<0.001
Positive ANA (*n*, %)	n. a.	92 (92%)	n. a.
Positive Anti ds-DNA (*n*, %)	n. a.	68 (68%)	n. a.
Serum MMP-9 (×10^2^ ng/L) [med (min–max)]	22 (10.4–30)	8 (1.1–37)	<0.001

Notes: Data are presented as mean SD, and [median (minimum-maximum)]. Student’s *t*-test and chi-square test were used. Abbreviations: *n*: number in each group, M: mean, n. a: not applicable, MMP-9: matrix metalloproteinase-9, [med (min–max)]: [median (minimum–maximum)]. *p* < 0.05 is considered significant when compared to the control group.

**Table 3:** Gelatinase B/MMP-9 rs3918249 T/C and rs17576 A/G alleles, and genotypes, both the SLE study participants and the healthy controls.

Genetic Model	Genotype	Controls	SLE	*p*	OR	95% CI
		(*n* = 100)	(*n* = 100)			
MMP-9 rs3918249 T/C						
Additive model	TT, *n* (%)	19(19%)	16(16%)	-	1	Reference
	TC, *n* (%)	40 (40%)	42 (42%)	0.586	1.148	0.699–1.887
	CC, *n* (%)	41 (41%)	42 (42%)	0.628	1.131	0.689–1.856
Dominant model	TC + TT, *n* (%)	81 (81%)	84 (84%)	0.577	1.139	0.721–1.801
Allelic model	T, *n* (%)	78 (39%)	74 (37%)	-	1	Reference
	C, *n* (%)	122 (61%)	126 (63%)	0.680	1.055	0.819–1.358
	HWE	0.111	0.322			
MMP-9 rs17576 A/G						
Additive model	AA, *n* (%)	46(46%)	55(55%)	-	1	Reference
	AG, *n* (%)	37(37%)	33(33%)	0.347	0.832	0.568–1.220
	GG, *n* (%)	17(17%)	12(12%)	0.215	0.719	0.427–1.211
Dominant model	AG + GG, *n* (%)	54(54%)	45(45%)	0.203	0.798	0.563–1.130
Allelic model	A, *n* (%)	129(64.5%)	143(71.5%)	-	1	Reference
	G, *n* (%)	71(35.5%)	75(28.5%)	0.134	0.817	0.627–1.064
	HWE	0.055	0.057			

Notes: Values are expressed as a percentile. *p* > 0.05 is considered non-significant. Test, Logistic regression analysis. Abbreviations: *n*: number in each group; OR: odds ratio; CI: confidence interval; HWE: the analyzed genotypes were in Hardy–Weinberg equilibrium (HWE); Freq: frequency, SLE: systemic lupus erythematosus, MMP-9: matrix metalloproteinase-9.

**Table 4:** Gelatinase B/MMP-9 rs3918249 T/C and rs17576 A/G haplotypes in the studied SLE patients and healthy control subjects.

Haplotype	rs3918249 T/C	rs17576 A/G	Controls (Frequency)	SLE Patients (Frequency)	*p*	OR	95% CI
1	T	A	0.059	0.254	-	1	-
2	C	A	0.586	0.461	<0.001	0.36	0.206–0.631
3	T	G	0.331	0.116	<0.001	0.226	0.119–0.429
4	C	G	0.024	0.169	0.405	1.473	0.592–3.664

Notes: *p* less than 0.05 is statistically significant. The HaploView program (version 4.2) was used to calculate the haplotypes. Abbreviations: OR: odds ratio; CI: confidence interval; SLE: systemic lupus erythematosus, MMP-9: matrix metalloproteinase-9.

**Table 5:** Gelatinase B/MMP-9 genotypes (rs3918249 T/C and rs17576 A/G) regression analysis for the prediction of clinical characteristics of systemic lupus erythematosus (SLE).

Clinical SLE Features	*n* (%)	Gelatinase B/MMP-9 (rs3918249 T/C)			*p* Values	Gelatinase B/MMP-9 (rs17576 A/G)			*p* Values
		TT (*n* = 16)	TC (*n* = 42)	CC (*n* = 42)		AA (*n* = 55)	AG (*n* = 33)	GG (*n* = 12)	
Skin	53 (53%)	9 (56.3%)	24 (57.1%)	20 (47.6%)	0.776	26 (47.3%)	17 (51.5%)	10 (83.3%)	0.205
Ulcers	10 (10%)	2 (12.5%)	3 (7.1%)	5 (11.9%)	0.721	3 (5.5%)	5 (12.2%)	2 (16.7%)	0.099
Alopecia	31 (31%)	2 (12.5%)	17 (40.5%)	12 (28.6%)	0.077	17 (30.9%)	10 (30.3%)	4 (33.3%)	0.983
Arthritis	27 (27%)	1 (6.3%)	12 (28.6%)	14 (33.3%)	0.088	12 (21.8%)	11 (33.3%)	4 (33.3%)	0.198
Serositis	3 (3%)	0 (0%)	1 (2.4%)	2 (4.8%)	0.999	1 (1.8%)	1 (3%)	1 (8.3%)	0.451
Neurologic	13 (13%)	0 (0%)	10 (23.8%)	3 (7.1%)	0.999	8 (14.5%)	3 (9.1%)	2 (16.7%)	0.611
Blood disorder	72 (72%)	11 (68.8%)	31 (73.8%)	30(71.4%)	0.754	41 (74.5%)	21 (63.6%)	10 (83.3%)	0.531
Nephritis	67 (67%)	8 (50%)	27 (64.3%)	32 (76.2%)	0.123	36 (65.5%)	22 (66.7%)	9 (75%)	0.716
SLEDAI %, [med (min–max)]	100 (100%)	8 (0–19)	9 (4–19)	10 (0–18)	0.452	8 (0–19)	8 (4–19)	10 (8–18)	0.705
Active SLE > 10	49(49%)	10 (62.5%)	21 (50.0%)	20 (47.6%)	0.316	29 (52.7%)	18 (54.5%)	4 (33.3%)	0.702
Inactive SLE < 10	51 (51%)	6 (37.5%)	21 (50.0%)	22 (52.4%)		26 (47.3%)	15 (45.5%)	8 (66.7%)	
S.MMP-9 %, [med (min–max)]	100 (100%)	10 (3.1–22)	7.5 (2.3–22.8)	8.35 (1.1–37)	0.721	8.6 (2.3–22.8)	9.2 (2–37)	5.6 (1.1–22)	0.792

Notes: Data in parentheses are percentages or data are presented as [median (minimum–maximum)]. Test, Chi-square for categorical data, Logistic regression analysis was used. Abbreviations: SLE: systemic lupus erythematosus, S.MMP-9: serum matrix metalloproteinase-9, SLEDAI: Systemic Lupus Erythematosus Disease Activity Index *p* > 0.05 is statistically non-significant.
